# Pharmacological treatment of idiopathic pulmonary fibrosis – preclinical and clinical studies of pirfenidone, nintedanib, and N-acetylcysteine

**DOI:** 10.3402/ecrj.v2.26385

**Published:** 2015-02-10

**Authors:** Marjukka Myllärniemi, Riitta Kaarteenaho

**Affiliations:** 1 Department of Pulmonary Medicine, Helsinki University Central Hospital, Heart and Lung Center and the University of Helsinki, Helsinki, Finland; 2 Department of Internal Medicine, Respiratory Diseases, Institute of Clinical Medicine, University of Oulu, Oulu, Finland; 3 Medical Research Center Oulu, Respiratory Research Unit, Oulu University Hospital, Oulu, Finland; 4 Unit of Medicine and Clinical Research, Pulmonary Division, University of Eastern Finland, Kuopio, Finland; 5 Division of Respiratory Medicine, Center for Medicine and Clinical Research, Kuopio University Hospital, Kuopio, Finland

**Keywords:** Idiopathic pulmonary fibrosis, nintedanib, pirfenidone

## Abstract

Three recent clinical trials on the pharmacologic treatment of idiopathic pulmonary fibrosis (IPF) mark a new chapter in the management of patients suffering from this very severe fibrotic lung disease. This review article summarizes the published investigations on the preclinical studies of three novel IPF drugs, namely pirfenidone, nintedanib, and N-acetylcysteine (NAC). In addition, the study protocols, differences, and the main findings in the recent clinical trials of these pharmacological treatments are reviewed. The strategy for drug development and the timeline from the discovery to the clinical use have been very different in these regimens. Pirfenidone was discovered in 1976 but only recently received approval in most countries, and even now its exact mechanism of action is unknown. On the contrary, nintedanib (BIBF1120) was identified in large drug screening tests as a very specific inhibitor of certain tyrosine kinases, but no published data on preclinical tests existed until 2014. NAC, a mucolytic drug with an antioxidant mechanism of action was claimed to possess distinct antifibrotic properties in several experimental models but proved to be ineffective in a recent randomized placebo-controlled trial. At present, no curative treatment is available for IPF. A better understanding of the molecular mechanisms of IPF as well as relevant preclinical tests including animal models and *in vitro* experiments on human lung cells are needed to promote the development of therapeutic drugs.

The positive results emerging from animal studies and phase I trials stimulated the initiation of several clinical trials in idiopathic pulmonary fibrosis (IPF) in 1990s and 2000s; however, most of them produced only disappointing results when they reached the randomized phase III studies (1). It was not until the present decade that the optimization of clinical study protocols with suitable endpoints started to yield clinically applicable results for the pharmacological treatment of IPF. Two recent trials have proved that the previously commonly used triple drug regimen, that is, the combination treatment of prednisone, azathioprine, and N-acetylcysteine (NAC) is harmful or at least ineffective in patients with IPF (2) and furthermore found no supportive evidence for the use of NAC as a monotherapy (3). In contrast, several trials at present have shown that pirfenidone and nintedanib can be efficient in preventing the decline in lung function in IPF patients (4–8). Pirfenidone, in addition to its inhibitory effects on the reduction of forced vital capacity (FVC) (5, 6), has also been shown to have a significant effect on the mortality of the IPF patients (7).

Although these trials mark a novel era in the clinical treatment of IPF, they also highlight the need for researcher-driven studies on drug efficacy as well as necessity of conduction comparative studies of different regimens of the patients representing various phenotypes of IPF. It would be crucial to clarify the experimental foundations of these molecules and their mechanisms of action to create a foundation for future basic and clinical studies. Since the cost of drug development is so enormous, it would be most advantageous if clinical researchers in the near future could develop markers of therapeutic effect, that is, not only for monitoring changes in disease severity but also as possible indicators for treatment cessation.

Figure 1 lists the hallmark studies on the development of three pharmacological treatments for IPF, namely pirfenidone, nintedanib, and NAC. The time frame of each regimen shows a marked variation from the point of discovery to their entry into clinical use. The preclinical studies on nintedanib were not published before the drug was evaluated in a clinical IPF phase III trial, since the first preclinical study on pulmonary fibrosis was conducted in 2007 with BIBF1000, a sibling molecule of nintedanib, that is, BIBF1120 (9). Nintedanib, that is, BIBF1120 was discovered as a side product from large screening assays targeting the cyclin-dependent kinase (CDK4) kinase (10, 11). Nintedanib was systematically developed by a pharmaceutical company (Boehringer Ingelheim) as a potent angiogenesis inhibitor. In contrast, NAC was discovered in the 1960s and there was a vast amount of preclinical data demonstrating inhibitory effects involving several antifibrotic mechanisms both *in vitro* and *in vivo*, but there was still a lack of firm conclusive evidence to support its clinical use in the treatment of IPF.

**Fig. 1:**
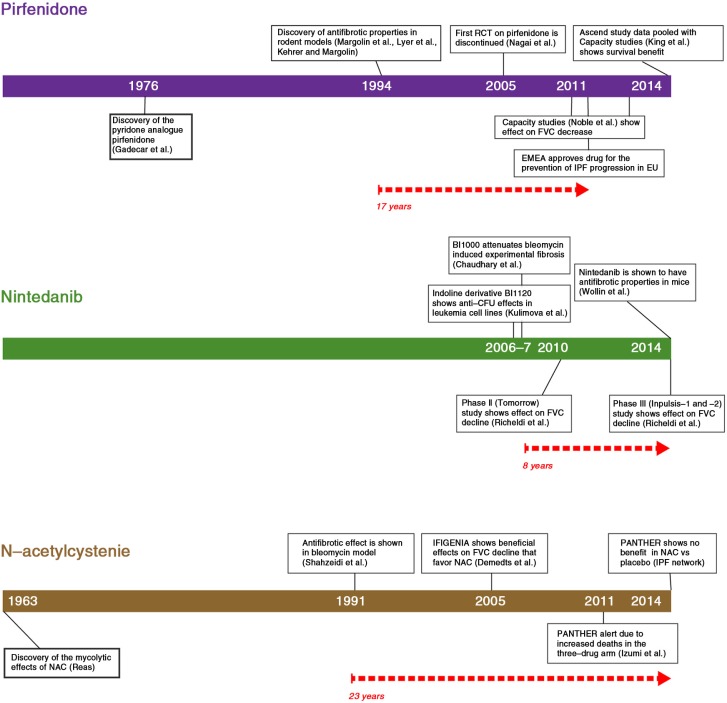
The timeline from discovery to clinical application of three IPF drugs. The pathway from preclinical discovery to clinical application varies from drug to drug. The development of NAC as an antifibrotic drug required nearly four decades but it did prove to be disappointing in the latest randomized clinical trial, whereas nintedanib had not been extensively tested in experimental animals even though it had been shown to accelerate the FVC decline in humans – only a few years after its initial discovery. The initiation of pirfenidone to clinical applications, on the other hand was delayed by several problems in trial design.

## Antifibrotic mechanisms of action of pirfenidone in lung fibrosis

The earliest studies on pirfenidone in lung fibrosis were mainly conducted in the 1990s by the same researchers using a bleomycin-induced pulmonary fibrosis model in hamsters. These studies indicated that pirfenidone could reduce the expression of several profibrotic factors in lung tissue and/or bronchoalveolar lavage (BAL) fluid. Pirfenidone has been shown to prevent the accumulation of hydroxyproline, procollagen I and III, inflammatory cells and transforming growth factor-beta (TGF-β) in BAL, and/or lung tissue (12–18). Later these findings were reproduced in other species (mice, cats) and different models (amiodarone and bleomycin) (19–22). Pirfenidone has also been shown to diminish the fibrocyte pool and the migration of these cells in the bleomycin-induced lung fibrosis model in mice (23). Recently, the results of cell culture experiments conducted *in vitro* on human lung fibroblasts cells have revealed that pirfenidone exerts many effects, that is, a decrease in fibroblast proliferation, reduction of TFG-β stimulated reactions, lowered levels of a myofibroblast marker alpha smooth muscle actin (α-SMA), and reduced expression of heat shock protein 47 (HSP47) (24, 25). A compilation of the preclinical studies on pirfenidone in lung fibrosis is shown in Table 1.

**Table 1:** Preclinical studies on pirfenidone in pulmonary fibrosis indicated by the name of the first author and the year of publication (reference number in brackets)

Study	Method	Results
Iyer 1995, 1998, 1999, 2000 (12–15); Gurujeyalakshmi 1999 (26); Schelegle 1997 (17); Mansoor 1999 (18)	Bleomycin – hamster	**↓** Lung hydroxyproline, fibrosis, MPO, SOD activity, procollagen I and III, inflammatory cells, PDGF isoforms, and TGF-β in BAL **↑** lung function
Giri 1999 (27)	*In vitro* studies	Scavenge reactive oxygen species
Card 2003 (19)	Amiodarone – hamster	**↓** Fibrosis
Spond 2003 (28)	Antigen challenge – mouse	**↓** Total cells, eosinophils, neutrophils, IL-6 in BAL
Kakugawa 2004 (20)	Bleomycin – mouse	**↓** Fibrosis, HSP47+ cells and myofibroblasts
Liu 2005 (29)	Lung transplant model – rat	**↓** Collagen, arginase, TGF-β, TGF-β stimulated arginase activity in tissue and fibroblast
Zhou 2005 (30)	OB-model – mouse	**↓** OB-lesions, TGF-β in plasma and tissue
Tian 2006 (21)	Bleomycin – rat	**↓** Collagen, TGF-β and TIMP-1
Nakayama 2008 (24)	Human lung fibroblast	**↓** HSP47 and collagen I in TGF-β-stimulated fibroblasts
Oku 2008 (22)	Bleomycin – mouse	**↓** Hydroxyproline, IFN-γ, bFGF, TGF-β
Triverdi 2012 (31)	Bleomycin – mouse Intratracheal	**↓** Hydroxyproline, BAL cellularity
Inomata 2014 (23)	Bleomycin – mouse	**↓** Fibrosis, fibrocyte pool size via attenuation CCC2 and CCL12, fibrocyte migration
Conte 2014 (25)	Primary human lung fibroblasts	**↓** Fibroblast proliferation, TGF-β-induced α-SMA and procollagen I

MPO, myeloperoxidaxe; SOD, superoxide dismutase; PDGF, platelet-derived growth factor; TGF-β, transforming growth factor-beta; IL-6, interleukin 6; BAL, bronchoalveolar lavage; HSP47, heath shock protein 47; OB, obliterative bronchiolitis; TIMP, tissue inhibitor of matrix metalloproteinase; IFN-γ, interferon gamma; FGF, fibroblast growth factor; Fn, fibronectin; CCC2 and CCL12, chemokines; α-SMA, alpha smooth muscle actin.

## Clinical trials on pirfenidone

The timeline of pirfenidone from the drug development and preclinical studies to the worldwide acceptance as the first drug of choice of IPF is shown in Figure 1. International (32) and Japanese (33) open-label studies initially described promising results in favor of pirfenidone. The drug was first studied in a randomized setting in Japan (4). The primary endpoints of the study, the lowest oxygen saturation by pulse oximetry SpO2 during a 6-min exercise test, were not achieved due to actions initiated by the Japanese drug authority. Based on an interim analysis at 6 months of a secondary endpoint, acute exacerbation, the authority recommended early termination of the trial on ethical grounds. Acute exacerbation of IPF was manifested in 14% of the placebo group (5/35) compared to zero patients in the pirfenidone group during the first 9 months. After these results, the recommendation to provide pirfenidone to all IPF patients in Japan was issued. Even though this trial and its premature cessation led to the acceptance of the use of pirfenidone for the treatment of IPF in Japan, other drug authorities felt that the evidence to support the use of pirfenidone was weak, and the drug was not approved for worldwide use. It was not until another randomized Japanese (5) study and the Capacity studies (6) published in 2011 demonstrating 30% reduction in FVC decline at 52 weeks in one of the two trials that the European Drug Authority (EMEA) approved the drug. Given that only one of two US studies was positive, the Federal Drug Administration (FDA) in the United States requested an additional placebo-controlled study to demonstrate the efficacy of pirfenidone in IPF. That particular study – Ascend – performed in collaboration with the FDA, finally confirmed the effect of pirfenidone to prevent the FVC decline (7). Furthermore, a pooled analysis of the Capacity and the Ascend studies revealed a positive outcome in terms of mortality. The overall mortality of the IPF patients in clinical trials has been shown to be low (34, 35); therefore, the presented results can be considered as highly significant.

## Development and preclinical studies on nintedanib in lung fibrosis

Nintedanib was discovered by Boehringer–Ingelheim during the development of a CDK4 kinase inhibitor project, where it was found to have a specific inhibitory profile against three tyrosine kinase receptors, namely platelet-derived growth factor receptor alpha (PDGFRα), several vascular endothelial growth factor receptors (VEGFR-1, -2, and -3), and fibroblast growth factor receptor 1 (FGFR-1) but *not* CDK4 (10). As a potential angiogenesis inhibitor, it has been widely studied in clinical phase I-II trials against several types of cancer, including gastrointestinal (36), gynecological (37), and breast cancer (38). Its efficacy in the treatment of non-small lung cancer has been recently shown (39). However, there are few experimental and animal studies on nintedanib, also known as BIBF1120, on lung fibrosis. BIBF1000 – a molecule from the same type of drug development process as nintedanib – was initially chosen as a preclinical candidate molecule for triple kinase inhibition (10), but it was soon replaced by BIBF1120, possibly because the latter compound had a sustained inhibitory profile to the VEGFRs or had an additional inhibitory effect on the Src-type kinases that was thought to be beneficial in acute myeloid leukemia (11). Irrespective of the reason for this change, preclinical fibrosis studies related to nintedanib were actually done with this rather similar molecule, BIBF1000. BIBF1000 was shown to decrease the accumulation of collagen and profibrotic gene expression as well as myofibroblast differentiation in bleomycin-induced rat lung and human lung fibroblasts *in vitro* (9). Only after the clinical studies had been published did Wollin and co-authors publish preclinical data on nintedanib. They revealed that nintedanib has an inhibitory effect both on PDGF receptors α and β (a finding which had not been previously reported); on fibroblast proliferation and fibroblast-myofibroblast transformation, inflammatory cell, and collagen accumulation; fibrosis and granuloma formation in mouse bleomycin- and silica-induced fibrosis models as well as primary human fibroblasts (40) (Table 2).

**Table 2:** Preclinical studies on nintedanib and BIBF 1000 in pulmonary fibrosis indicated by the name of the first author and the year of publication (reference number in brackets)

Study	Method	Results
Chaudhary 2007 (9)	Bleomycin – rat Primary human lung fibroblasts from IPF, sarcoidosis and normal lung	**↓** Level of fibrosis, procollagen I, fibronectin, CTGF in tissue **↓** TGF-β-induced α-SMA in lung cells
Wollin 2014 (40)	Bleomycin-mouse Silica-mouse Human lung fibroblast	**↓** PDGF receptor activation, fibroblast proliferation, TGF-β induced α-SMA gene in cells **↓** Lymphocytes, neutrophils, IL-Iβ, TIMP-1 in BAL **↓** Collagen, inflammation, fibrosis, and granuloma formation in tissue

IPF, idiopathic pulmonary fibrosis; CTGF, connective tissue growth factor; TGF-β, transforming growth factor beta; α-SMA, alpha smooth muscle actin; PDGF, platelet-derived growth factor; IL-1β, interleukin-1 beta; TIMP, tissue inhibitor of matrix metalloproteinase; BAL, bronchoalveolar lavage.

## Clinical trials on nintedanib in lung fibrosis

Promising results on nintedanib on fibrosis progression were reported from a phase II randomized study using two different dosages of nintedanib (41). With the higher dosage of 150 mg twice daily, nintedanib displayed a trend in slowing the decline in FVC. In addition, a lower incidence of acute IPF exacerbations was also observed. These results raised high expectations in a second drug for the treatment of IPF and these were met with the completion of the two phase III studies on 1066 IPF patients, Inpulsis-1 and Inpulsis-2 (8). In the Inpulsis-2 trial, there was a decrease in the incidence of acute exacerbations. Overall, the incidence of acute exacerbations was low, 7.6% in the placebo group and 4.9% in the nintedanib group, suggesting that the study was not powerful enough to detect effects on acute exacerbations. Similarly, no effect on mortality was observed, even though trends to favor nintedanib were seen.

## Preclinical trials on NAC

The mucolytic actions of NAC were discovered in 1963 and it was primarily used as a mucolytic in the treatment cystic fibrosis (42). Several decades later a study on bleomycin-induced lung fibrosis in rats showed that NAC inhibited collagen accumulation in the lung (43). In the 1990s, investigators analyzed the efficacy of the short-term treatment of NAC in patients with various types of pulmonary fibrosis, IPF, and sarcoidosis, and noted that NAC increased glutathione in BAL fluid of the patients (44, 45). In bleomycin-induced fibrosis models of rats and mice, NAC was observed to inhibit several profibrotic mechanisms such as the amounts of hydroxyproline, collagen, fibrosis, several cytokines, inflammatory cells, mucus secretory cells, and mucin subtype 5ac (MUC5ac) (46–49). NAC has been shown to inhibit epithelial–mesenchymal transition (EMT) in rat alveolar epithelial cells (50), to diminish TGF-β-induced gel contraction, fibronectin (Fn), and VEGF production as well as α-SMA expression in human lung fibroblasts (51) as well as the concentrations of several cytokines produced by alveolar macrophages of IPF patients (52, 53). Recent animal studies on NAC with bleomycin- or silica-models have revealed a reduction in the fibrosis score, protection against lung injury, and a decrease in the reactive oxygen species’ (ROS) content in alveolar macrophages (54–56). The preclinical studies with NAC conducted in pulmonary fibrosis are summarized in Table 3.

## Clinical trials on NAC

Although experimental data (Table 3) and a large set of clinical open-label studies on the antifibrotic effects of NAC were very convincing, there is only one placebo-controlled trial on the efficacy of NAC monotherapy for the treatment of IPF (the Panther-IPF study (2, 3)). Initial clinical reports on the efficacy of NAC were based on the Ifigenia study, where a three-drug regimen (prednisone, azathioprine, and NAC) was reported to be more efficient than the two-drug therapy (prednisone and azathioprine) that was being used conventionally to treat IPF (60). However, the placebo-controlled Panther-IPF study revealed no positive effects on the study endpoints (3). The NAC-treated patients exhibited surprisingly few side effects, for example, no gastrointestinal side effects were reported. The Panther-IPF study was initially started with three arms: 1) three-drug regimen (NAC, prednisolone, and azathioprine), 2) NAC + placebo, and 3) placebo. An interim analysis of this study (2) performed at midpoint (30 weeks) of the study led to a recommendation to discontinue the three-drug regimen immediately as it had increased mortality (8 vs. 1) and serious adverse events (24 vs. 8), respectively, compared to the placebo group. For this reason, the entire study was interrupted for 3 months but later continued enrolling more patients into the two remaining arms (NAC vs. placebo, altogether 264 patients). It is possible that the relatively small sample size and the interruption of the study affected the study outcome. The results presented so far do not provide any support for the clinical use of NAC in the treatment of IPF.

**Table 3:** Preclinical studies on N-acetylcysteine in pulmonary fibrosis indicated by the name of the first author and the year of publication (reference number in brackets)

Study	Methods	Results
Shahzeidi 1991 (43)	Bleomycin – rat	**↓** Collagen in lung
Meyer A, 1994 (44)	17 IPF patients (5 days oral NAC)	**↑** Glutathione in BAL
Meyer 1995 (57)	8 PF, 6 control patients (iv NAC, 3 h)	**↑** Glutathione levels in IPF, not in controls
Behr 1997 (45)	18 IPF patients (12 wk oral NAC)	**↓** Met **↑** GSH in BAL **↑** Pulmonary function
Hagiwara 2000 (46)	Bleomycin – mouse (inhaled NAC)	**↓** Cellularity in BAL + tissue, hydroxyproline, fibrosis, several cytokines
Cortijo 2001 (47)	Bleomycin – rat	**↓** Collagen and inflammatory cells in tissue (but not cells in BAL), **↑** GSH in BAL
Serrano-Mollar 2003 (48)	Bleomycin – rat	**↓** Collagen, inflammation
Mata 2003 (49)	Bleomycin – rat	**↓** MUC5ac protein + mRNA, collagen, fibrotic area, TNF-α, MPO activity, mucus secretory cells
Felton 2009 (50)	Rat alveolar epithelial cells	**↓** EMT
Sugiura 2009 (51)	Human fetal lung fibroblasts (HFL-1)	**↓** TGF-β-augmented gel contraction, Fn + VEGF production, TGF-β-stimulated α-SMA
Cu 2009 (58)	Alveolar macrophages from 16 IPF patients	**↓** TNF-α, its receptors, TGF-β + LPS-stimulation and IL-1
Radomska-Leśniewska 2010 (52)	Alveolar macrophages from 4 IPF and 5 sarcoidosis patients	**↓** IL-8, MMP-9, and ICAM
Patel 2012 (53)	Bleomycin	**↓** EC alteration
Li 2012 (59)	Bleomycin – rat	**↓** Lox activity via elevation of GSH
Wang 2013 (54)	Bleomycin – mouse NAC pretreated human embryonic mesenchymal stem cells	Protects against lung injury
Zhang 2013 (55)	Silica – rat	**↓** Fibrosis score, HYP, MDA, TNF-α, IL-8 and hsCRP in BAL + serum
Zhang 2014 (56)	Silica – rat	**↓** ROS content of AM, mitochondrial apoptosis

BAL, bronchoalveolar lavage; IPF, idiopathic pulmonary fibrosis; GSH, glutathione; MUC5ac, mucin subtype 5ac; TNF-α, transforming growth factor alpha; MPO, myeloperoxidase; EMT, epithelial mesenchymal transition; TGF-β, transforming growth factor beta; Fn, fibronectin; VEGF, vascular endothelial growth factor; α-SMA, alpha smooth muscle actin; LPS, lipopolysaccharide; IL-8, interleukin 8; MMP-9, matrix metalloproteinase 9; ICAM, intracellular adhesion molecule; EC, endothelial cell; Lox, lysyl oxidase; HYP, hydroxyproline; MDA, malondialdehyde; hsCRP, high-sensitivity C-reactive protein; ROS, reactive oxygen species; AM, alveolar macrophage; iv, intravenous; wk, week; MET, methionine sulfoxide content.

## Differences in trial design and results

The enrolment criteria of the two randomized trials with a positive outcome, that is, studies on pirfenidone (Ascend) and nintedanib (Inpulsis) are shown in Table 4. Even though the study protocols share many similar features in terms of follow-up time (52 weeks) and primary endpoints (FVC decline), there are also major dissimilarities that complicate the comparison of the study populations. One of the most important differences is found in the enrolment criteria, since the Ascend study excluded smokers and patients with obstruction and severe emphysema, whereas in the Inpulsis studies these exclusion criteria were not applied. In addition, the Inpulsis studies probably consisted of a more heterogeneous population of patients, which included patients without honeycombing, since possible usual interstitial pneumonia (UIP) pattern in high-resolution computed tomography (HRCT) according to the current guidelines have been included without a surgical lung biopsy. Nonetheless, the results of both trials showed a clear reduction in the rate of lung function decline. It is unfortunate that the endpoints cannot be directly compared, as the missing data (dropouts or deaths) have been computed differently in the two trials (Table 5). The Ascend trials assigned the worst rank or outcome (i.e. FVC 0, when data are missing), but the Inpulsis and Panther trials have simply not accounted for missing data. In the Inpulsis, very few patients actually dropped out of the trial. This difference in statistical analysis makes any direct comparison of placebo groups in the two trials very difficult. The endpoints and main results of the randomized pirfenidone, nintedanib, and NAC studies are shown in Table 5.

**Table 4:** Enrolment criteria in the Ascend and Inpulsis trials

Inclusion criteria	Ascend	Inpulsis
Age	40–80	>40
IPF diagnosis	Centrally confirmed diagnosis. Clinical symptoms consistent with IPF>12 months duration. Diagnosis of IPF 6–48 months before randomization.	IPF diagnosis within previous 5 years
Lung function	FVC 50–90% DLCO 30–90% FEV1/FVC≥0.80	FVC≥50% DLCO 30–79% FEV1/FVC<0.7 excluded
HRCT	HRCT: definite UIP, or possible UIP+surgical lung biopsy (SLB) confirmation Extent of fibrotic changes (honeycombing, reticular changes) greater than extent of emphysema on HRCT scan	HRCT criteria if a SLB was not available: A+B+C, or A+C, or B+C A=definite honeycomb lung destruction with basal and peripheral predominance B=presence of reticular abnormality and traction bronchiectasis consistent with fibrosis with basal and peripheral predominance C=atypical features are absent, specifically nodules and consolidation. Ground glass opacity, if present, is less extensive than reticular opacity pattern
Surgical lung biopsy (SLB)	Definite UIP, probable UIP or possible UIP in SLB+definite or possible UIP in HRCT according to the guidelines of 2011 ATS/ERS SLB: 30.9% (placebo 28.5%)	Definite UIP, probable UIP, possible UIP, definitely not UIP SLB: INPULSIS-1, 19.4% (placebo 16.2%), INPULSIS-2, 25.5% (placebo 23.7%)
6MWT	6MWT 150 m or more	
Other treatment	Concomitant treatment with any investigational therapy was prohibited	Concomitant therapy with up to 15 mg of prednisone permitted if the dose had been stable for 8 or more weeks. Other IPF drugs excluded. After 6 months’ treatment, patients whose condition had deteriorated could receive azathioprine, cyclophosphamide, cyclosporine, NAC, or more than 15 mg of prednisone.
Smoking	Smoking within 3 months of screening (exclusion criteria)	Smokers included

HRCT, high-resolution computed tomography; 6MWT, 6-minute walking test; NAC, N-acetylcysteine; UIP, usual interstitial pneumonia; FVC, forced vital capacity; DLCO, diffusion capacity; FEV1, forced expiratory volume in 1 second.

**Table 5:** Study endpoints, differences in missing data inputation and main results in the most recent randomized trials on pirfenidone, nintedanib and N-acetylcysteine

Study	Primary endpoint	Main secondary endpoints	Missing data	Main result
Capacity-1 -2	Change % predicted FVC to week 72	Progression-free survival, dyspnoea 6MWT distance, worst (SpO2) during the 6MWT, DLco, HRCT	Missing values due to death were assigned the worst rank or outcome	Reduced mean decline in FVC % (pred)
Ascend	Change (baseline – week 52) in % Pred FVC	6-MWT (m) Risk of death UCSD SOBQ	Missing values due to death assigned worst rank or outcome	Pooled (*N*=1,247) risk of death reduction 48%
Inpulsis	FVC annual rate of decline from baseline	Time to the first acute exacerbation, decline in SGRC, rate of death	Missing data not imputed for the primary analysis^a^	Reduction of FVC decline, delay in acute exacerbations (Inpulsis-2)
Panther	FVC decline at week 60		Primary endpoint; not computed	No effect on FVC decline

a Data collected after discontinuation of the study drug was used in the primary analysis.

DLco, diffusing capacity; FVC, forced vital capacity; HRCT, high-resolution computed tomography; UCSD SOBQ, University of California Shortness of Breath Questionnaire; 6MWT, 6-minute walking test; SpO2, peripheral capillary oxygen saturation; SGRC, St. George's respiratory questionnaire.

## Discussion

For the first time in the history of the clinical research of the pharmaceutical treatment on IPF, the primary endpoints of several studies have revealed positive results. Even though the reduction of FVC decline by 30% did not exceed the hopes of clinicians and patients, these results are still promising since they raise the possibility of a prolonged survival for IPF patients. The three recently published studies, namely Ascend, Inpulsis, and Panther clearly showed that randomized and placebo-controlled multicenter trials are the most appropriate way to reveal clinical benefits of pharmacological treatment of IPF. The results of all these abovementioned studies will affect daily clinical practice not only through positive results of pirfenidone and nintedanib but also with the negative results obtained with NAC.

In view of the long history of preclinical and clinical investigations of NAC and pirfenidone, it can be concluded that it requires a long time to develop a drug from its discovery till its clinical application. On the contrary, the time period to the clinical use of nintedanib has been markedly shorter. Thus, there are some elements that need to be considered when future clinical trials are evaluated. First, it seems that interim analyses have sometimes broken study liability. In the first randomized pirfenidone trial (2), the decrease of acute exacerbations in the treatment arm led to study discontinuation, which ultimately led to a long delay before worldwide acceptance of the drug. In the Panther trial, it could be speculated that the discontinuation of one arm of the trial may have affected the results of the remaining placebo- and NAC monotherapy arms (3). The more recent studies on pirfenidone and nintedanib have not permitted interim analyses from their study protocols. Second, the applicability of preclinical data on IPF drugs must be viewed with caution due to at the high number of the studies claiming evidence on the efficacy for NAC as an antifibrotic agent whereas the endpoints of clinical placebo-controlled study were negative. In contrast, practically no preclinical data on nintedanib was published before it entered into clinical investigations resulting in positive results in phase II and III studies.

The preclinical investigations of all three regimens (NAC, pirfenidone, and nintedanib) reveal surprisingly similar antifibrotic mechanism of actions, namely decreased amounts of histological fibrosis, decreased tissue levels of collagen and/or hydroxyproline, and decreased levels of TGF-β and α-SMA. Each drug has, in addition, displayed several specific antifibrotic properties. A glutathione deficiency in epithelial lining fluid of the patients with IPF was observed several decades ago (61) but the evidence for the regulatory capability of glutathione has been mainly observed in the studies investigating NAC. Several *in vivo* studies have indicated that NAC can increase the levels of glutathione in BAL fluid of patients with IPF (57). In addition to the experiments from animal models, nintedanib and pirfenidone, but not NAC, have been investigated in human lung cell lines, revealing that both drugs are capable of diminishing fibroblast proliferation. NAC has been shown to regulate MUC5ac and epithelial EMT, phenomena that have not been investigated with either pirfenidone or nintedanib (49, 50). There are several important problems regarding preclinical studies on IPF, for example, the fact that none of the current animal models properly mimic the heterogeneous process of fibrosis in IPF, and that the pathogenetic mechanisms are still poorly understood. Even though this has been an area of active research on IPF in the past decade, we are still at the early stages of unravelling the etiopathogenesis of IPF (62).

Careful evaluation of the design of the future clinical studies will be necessary, as IPF is such a rare disease. The results of recent clinical trials raise many questions, for example, will it be possible to perform clinical trials in the future with sufficient numbers of patients on trials investigating a novel drug versus pirfenidone, and moreover, will placebo-controlled trials still be approved by the authorities? How about the patients that do not fulfill the current IPF criteria or are at a very early or an advanced stage of the disease? It would probably be useful if future clinical trials could be performed using uniform inclusion criteria and have similar endpoints to allow data pooling and comparison between studies. In addition, more specific tools for the diagnostics of acute exacerbations would be helpful for providing more diverse endpoints. Most importantly, even though some important progress has been made, clinical studies must be continued, and novel drugs will still need to be investigated in a RCT setting. Furthermore, the patients with other types of fibrotic interstitial lung diseases are still lacking clinical research advances.

## Conclusion

Although major progress has been achieved in the clinical research into IPF, a curative therapy for this severe lung disease is still lacking, which emphasizes the need for all kinds of research, not simply clinical trials in IPF. Before one can develop novel pharmacological therapies to cure IPF, we will need innovative approaches, including basic and translational techniques, and most importantly, relevant animal models as well as protocols using human cells. If it were possible to provide respiratory physicians new tools for the treatment of the IPF patients, this in turn could advance further clinical research, for example, identifying those patients that will benefit most from the new therapeutic agents.
